# Toward understanding the fast latex coagulation in *Campanula* spp. (Campanulaceae)

**DOI:** 10.1093/iob/obaf020

**Published:** 2025-05-14

**Authors:** M H M Wermelink, M L Becker, R Konradi, C Taranta, M Ranft, S Nord, J Rühe, T Speck, S Kruppert

**Affiliations:** Plant Biomechanics Group @ Botanical Garden, University Freiburg, 79104 Freiburg im Breisgau, Germany; Excellence Cluster LivMatS, University Freiburg, 79110 Freiburg im Breisgau, Germany; Plant Biomechanics Group @ Botanical Garden, University Freiburg, 79104 Freiburg im Breisgau, Germany; Joint Research Network on Advanced Materials and Systems (JONAS), Freiburg Materials Research Center, University Freiburg, 79098 Freiburg im Breisgau, Germany; BASF SE, Group Research, Joint Research Network on Advanced Materials and Systems (JONAS), 67056 Ludwigshafen, Germany; BASF SE, Agricultural Solutions, 67117 Limburgerhof, Germany; BASF SE, Group Research, Joint Research Network on Advanced Materials and Systems (JONAS), 67056 Ludwigshafen, Germany; BASF SE, Agricultural Solutions, 67117 Limburgerhof, Germany; Excellence Cluster LivMatS, University Freiburg, 79110 Freiburg im Breisgau, Germany; Joint Research Network on Advanced Materials and Systems (JONAS), Freiburg Materials Research Center, University Freiburg, 79098 Freiburg im Breisgau, Germany; IMTEK, University Freiburg, 79110 Freiburg im Breisgau, Germany; Plant Biomechanics Group @ Botanical Garden, University Freiburg, 79104 Freiburg im Breisgau, Germany; Excellence Cluster LivMatS, University Freiburg, 79110 Freiburg im Breisgau, Germany; Joint Research Network on Advanced Materials and Systems (JONAS), Freiburg Materials Research Center, University Freiburg, 79098 Freiburg im Breisgau, Germany; Plant Biomechanics Group @ Botanical Garden, University Freiburg, 79104 Freiburg im Breisgau, Germany; Excellence Cluster LivMatS, University Freiburg, 79110 Freiburg im Breisgau, Germany; Joint Research Network on Advanced Materials and Systems (JONAS), Freiburg Materials Research Center, University Freiburg, 79098 Freiburg im Breisgau, Germany

## Abstract

The plant most commonly known for producing latex is the Pará rubber tree, *Hevea brasiliensis*. There are, however, thousands of latex-bearing plant species, and these species exhibit a diverse array of different types of latex, each type in accordance with its producers’ main selective pressure after injury. One key function of latex is to seal, but the most crucial necessities for wound sealing differ by the environment. For species growing in arid climates, for example, minimizing water loss is crucial whereas in tropical ecosystems a strong (chemical) defense against herbivores, parasites, and germs is of more imminent importance. This diversity of ecosystems and species’ environments is mirrored by a respective diversity in latices’ chemical compositions, material properties, and coagulation times. While some plant species solely rely on evaporation of water for their latex coagulation, the *H. brasiliensis* latex contains the coagulation assisting protein Hevein, allowing for coagulation in 30 min. With coagulation times of 10 s and below, species of the genus *Campanula* pose considerable challenge to the measurement of latex characteristics. We here present an overview to the coagulation of latex in the genus *Campanula* and reveal substantial differences to the latex coagulation of *H. brasiliensis*. For a collection of 6 different *Campanula* species, we determined coagulation times under different temperatures, latex dry weights, contact angles of water droplets on latex surfaces and imaged laticifer cross-sections using cryo-SEM. We found *Campanula* latex to coagulate significantly faster than *Hevea* latex and no evidence of *Hevea-*like lutoids in the laticifers. A coagulation test in a pressure chamber further revealed *Campanula* latex to coagulate at pressures of 8 bar, where latex coagulation in *Ficus benjamina*, which is described to have similar coagulation mechanism as *Hevea*, has previously been reported to be impaired. Our findings thus suggest *Campanula* latex coagulation to follow a different mechanism than the one described in *Hevea*.

## Nederlands

De meest bekende latexproducerende plant is de Braziliaanse rubberboom, *Hevea brasiliensis*. Naast *Hevea* zijn er nog duizenden latexhoudende plantensoorten, die onderling een grote diversiteit aan latexen produceren. Elk type latex weerspiegelt de voornaamste selectriedruk op de respectievelijke soort na een verwonding. Een belangrijke functie van latex is wondsluiting, maar de noodzaak voor wondsluiting hangt af van de habitat van de plant. Voor soorten die groeien in bijvoorbeeld een droog klimaat, is het minimaliseren van waterverlies cruciaal, terwijl in tropische ecosystemen een sterke (chemische) verdediging tegen herbivoren, parasieten en bacteriën van groter belang is. Deze diversiteit aan ecosystemen en habitats van soorten wordt weerspiegeld in een respectievelijke diversiteit in de chemische samenstelling, materiaaleigenschappen en coagulatietijden van latex. Terwijl het latex van sommige plantensoorten coaguleert enkel door verdamping van water, bevat het latex van *H. Brasiliensis* het coagulatiebevorderende eiwit heveïne, wat coagulatie binnen 30 minuten mogelijk maakt. Met coagulatie in 10 seconden of minder vormen soorten van het geslacht *Campanula* een aanzienlijke uitdaging voor het meten van latexkenmerken. Met dit onderzoek geven wij een overzicht van de coagulatie van latex in het geslacht *Campanula* en onthullen substantiële verschillen met de latexcoagulatie van *H. Brasiliensis*. Voor een collectie van 6 verschillende campanulasoorten hebben we de coagulatietijden bij verschillende temperaturen, het drooggewicht van het latex en de contacthoeken van waterdruppels op latexoppervlakken gemeten. Daarnaast hebben we dwarsdoorsneden van laticifers vastgelegd met behulp van cryo-SEM. In onze experimenten coaguleerde *Campanula* latex significant sneller dan *Hevea* latex. Onze cryo-SEM beelden toonden geen *Hevea*-achtige lutoïden in de latificers van *Campanula*. Een coagulatietest bij hoge luchtdruk toonde tevens aan dat *Campanula* latex coaguleert bij een druk van 8 bar. Deze luchtdruk is gemeld de latexcoagulatie te verstoren in *Ficus benjamina*, waarvan het coagulatiemechanisme beschreven is als vergelijkbaar met dat van *Hevea*. Onze bevindingen suggereren dat de coagulatie van *Campanula* latex een ander mechanisme volgt dan welke beschreven is voor *Hevea*.

## Français

La plante la plus connue pour produire du latex est l'arbre à caoutchouc, *Hevea brasiliensis*. Cependant, il existe des milliers d'espèces de plantes qui présentent une grande variété de types de latex différents, chacun étant relié à la pression sélective principale de leurs producteurs après lésion. Une des fonctions clés du latex est protectrice, les nécessités les plus cruciales pour l'obturation des plaies diffèrent selon l'environnement. Pour les espèces qui poussent dans un climat aride, par exemple, la minimisation de la perte d'eau est cruciale, tandis que dans les écosystèmes tropicaux, une défense chimique forte contre les herbivores, les parasites et les germes est plus importante. Cette diversité d'écosystèmes et d'environnements des espèces se reflète dans une diversité respective de la composition chimique, des propriétés matérielles et des temps de coagulation du latex. Alors que certaines espèces de plantes ne dépendent que de l'évaporation de l'eau pour la coagulation de leur latex, le latex de *H. brasiliensis* contient la protéine Hevein qui aide à la coagulation, permettant ainsi une coagulation en 30 minutes. Avec des temps de coagulation de 10 secondes et moins, les espèces du genre *Campanula* posent un défi considérable pour la mesure des caractéristiques du latex. Nous présentons ici une vue d'ensemble de la coagulation du latex dans le genre *Campanula* et révélons des différences substantielles avec la coagulation du latex de *H. brasiliensis* : pour une collection de 6 espèces différentes de *Campanula*, nous avons déterminé les temps de coagulation sous différentes températures, les poids secs du latex, les angles de contact des gouttes d'eau sur les surfaces de latex et imager les sections transversales des laticifères à l'aide de la cryo-SEM. Nous avons constaté que le latex de *Campanula* coagule significativement plus vite que le latex d'*Hevea* et qu'il n'y a pas de preuve de la présence de lutoides de type *Hevea* dans les laticifères. Un test de coagulation dans une chambre à pression a révélé en outre que le latex de *Campanula* coagule à des pressions de 8 bar, alors que la coagulation du latex de *Ficus benjamina*, qui est décrite comme ayant un mécanisme de coagulation similaire à celui d'*Hevea*, a été précédemment signalée comme étant altérée. Nos résultats suggèrent ainsi que la coagulation du latex de *Campanula* suit un mécanisme différent de celui décrit pour *Hevea*.

## Deutsch

Die wohl bekannteste latexproduzierende Pflanze ist der Pará-Kautschukbaum (*Hevea brasiliensis*). Neben *Hevea* gibt es jedoch Tausende von latexhaltigen Pflanzenarten, welche eine Vielzahl unterschiedlicher Latizes produzieren. Die jeweilige Latexzusammensetzung und die Art der Koagulation nach einer Verletzung korreliert mit den dominierenden Selektionsdrucken im jeweiligen Ökosystem. Eine wichtige Funktion des Latex ist der Wundschluss, die Notwendigkeit für den Wundschluss ist allerdings unterschiedlich für verschiedene Lebensräume. Für Arten, die z.B. in einem ariden Klima wachsen, ist die Minimierung des Wasserverlustes entscheidend, während in tropischen Ökosystemen eine starke (chemische) Verteidigung gegen Pflanzenfresser, Parasiten und Keime von größerer Bedeutung ist. Diese Vielfalt der Ökosysteme und der Lebensräume der Arten spiegelt sich in einer entsprechenden Vielfalt der chemischen Zusammensetzung, der Materialeigenschaften und der Koagulationszeiten der Latizes wider. Während einige Pflanzenarten für die Gerinnung ihres Latex ausschließlich auf die Verdunstung angewiesen sind, enthält der Latex von *H. brasiliensis* das koagulationsfördernde Protein Hevein, das eine Koagulation innerhalb von 30 Minuten ermöglicht. Mit Koagulationszeiten von 10 Sekunden und weniger stellen die Arten der Gattung *Campanula* dagegen erhebliche Herausforderung für die Messung der Latexeigenschaften dar. Wir geben in dieser Studie einen Überblick über die Latexkoagulation bei der Gattung *Campanula* und zeigen wesentliche Unterschiede zur Latexkoagulation von *H. brasiliensis* auf. Für eine Auswahl von 6 verschiedenen *Campanula*-Arten haben wir die Koagulationszeiten bei unterschiedlichen Temperaturen, das Trockengewicht des Latex und den Kontaktwinkel von Wasser auf der Latexoberfläche gemessen, sowie Querschnitte der Milchröhren angefertigt und mittels Cryo-SEM visualisiert. In unseren Versuchen koagulierte *Campanula*-Latex deutlich schneller als *Hevea*-Latex. Die Cryo-SEM Aufnahmen ergaben keine Hinweise für *Hevea*-ähnliche Lutoide in den Milchröhren von *Campanula*. Ein Koagulationstest in einer Druckkammer zeigte außerdem, dass *Campanula*-Latex bei einem Druck von 8 bar koaguliert, während die Latexkoagulation bei *Ficus benjamina* bei ähnlichem Druck beeinträchtigt ist. Der Koagulationsmechanismus bei *Ficus* ähnelt dem von *Hevea*. Unsere Ergebnisse deuten darauf hin, dass die Koagulation von *Campanula*-Latex einem anderen Mechanismus folgt als dem für *Hevea* beschriebenen.

## Introduction

Predation is one of the major players in species interactions and consequently a strong driving force in evolution. While animals have the chance of avoiding predatory injuries through movement (e.g., flight response), plants are often limited to defensive strategies like thorns, spines, and toxins. Despite such apparent differences between animal and plant prey response options, all organisms have evolved the ability to self-repair. The level of possible self-repair differs between taxa but seems independent of the organism's level of complexity (for a review see [Harrington et al. 2016; Speck and Speck 2019]). Self-repair is crucial for the survival of a species in context of evolution as well as for the individual organism. Tissue damages that necessitate self-repair (i.e., wounds) are not only inflicted by predator-prey-interactions but can also derive from inter- and intraspecific competition, caused by the non-living environment (e.g., weather events) or happen during reproduction, e.g., during pollination or diaspore dispersal. Given the usual lack of movement capabilities, it appears to be even more important for plants to have reliable self-repair systems in order to control for predator-inflicted tissue damage. Successful healing undergoes distinguishable, consecutive phases from sealing the wound to regrowth of new tissue (Speck et al. 2013; Speck and Speck 2019). In plants, different sealing agents have evolved and often times are combined with chemical self-defense. In conifers, for example, resins have evolved that seal off a wound, trapping smaller herbivores and protecting the plant tissue from pathogens and parasites due to a combination of different toxic substances (Dell and McComb 1979; Langenheim 1990; Shuaib et al. 2013). Like resins, latices serve as protection against herbivores, pathogens, and parasites, but differ from resins in their chemical composition and the way of storage within the respective plant tissue. Resins, mainly rich in terpenoids and phenolics, are stored in intercellular spaces called resin ducts, while latex is stored in elongated specialized cells known as laticifers and consist mainly of rubber, proteins and alkaloids (Dell and McComb 1979; Langenheim 1990; Agrawal and Konno 2009; Konno 2011; Foisy et al. 2019). Latex has evolved independently multiple times, and is found today in numerous plant species from various evolutionary lineages (Metcalfe 1967; Metcalfe 1983; Farrell et al. 1991). The most commonly known latex is the one from *Hevea brasiliensis*, the Pará rubber tree. Latex from *H. brasiliensis* is broadly used in industry, well-studied, and its coagulation is understood in detail (Gidrol et al. 1994; Ng et al. 2022). Other latex bearing plants, however, are not as intensively studied but bear interesting features, as every latex evolved to thwart the very species' ecological challenges. Different climates, for example, can cause different functions of a plant's latex to be prioritized over others. While in tropical climates the chemical defense aspects of latex are crucial to counter a high diversity and abundance of herbivores, in arid climates a fast and reliable wound-sealing is imperative to minimize water loss. Consequently, latices differ in their chemical composition and physical properties as much as the ecology of the plant species producing them (Metcalfe 1967; Farrell et al. 1991; Lewinsohn 1991; Gidrol et al. 1994; Konno 2011; Sytwala et al. 2015; Hua et al. 2017; Ramos et al. 2019; Ng et al. 2022). In tropical regions the number of laticiferous plants appears to be significantly higher (14% of all tropical species) than in temperate climate (6% of all temperate species) (Farrell et al. 1991). This larger proportion of laticiferous plants in tropical regions may be a result of predation pressure from a diverse group of herbivores. Well studied examples of tropical laticiferous plants with effective herbivore defense include *Carica papaya* (Konno et al. 2004) as well as *H. brasiliensis* (Van Parijs et al. 1991; Kanokwiroon et al. 2008).

While the defensive functions of latices have been studied and described through ecological and toxicological studies, the coagulation mechanisms are still largely unknown. One exception is the Pará rubber tree *H. brasiliensis* whose latex coagulation mechanism is described in detail (Gidrol et al. 1994; Ng et al. 2022). The coagulation of *H. brasiliensis* latex takes about 30 min and is a complex series of physicochemical processes: In the laticifers, latex particles are dispersed as a colloid which is stabilized by electrostatic repulsion between these charged particles. The laticifers in *H. brasiliensis* are under a pressure of over 8 bar which, in case of injury, drops immediately to ambient pressure (Buttery and Boatman 1966). This sudden decrease in pressure causes vesicles in the latex, so called lutoids, which store Ca^2+^ ions as well as a protein named Hevein, to burst open. Both the Ca^2+^ ions and Hevein aid the latex particles in connecting to each other and thus speed up the process of coagulation, that is, the merging of the latex particles (Gidrol et al. 1994; Ng et al. 2022). The latex of the genus *Ficus* coagulates using the same components and mechanisms as that of *Hevea* but with a coagulation time of 20 min it is even faster (Bauer et al. 2010). In contrast to the coagulation mechanism described in *Hevea* and *Ficus*, members of the genus *Euphorbia* have been found to rely solely on water evaporation in order to let their latex particles merge and coagulate (Bauer et al. 2014a, 2014b). There is a genus of plants, however, that bears latex coagulating remarkably faster than any of the aforementioned species: the bellflower (*Campanula*). Latex of the genus *Campanula* has been reported to coagulate within seconds, posing severe challenges to analyzing its characteristics (Bauer et al. 2014b). We here present a comparison of coagulation time between different *Campanula* species as well as some characteristics of selected species’ latices including dry mass, wettability and a spectrophotometric analysis. The chosen methods either allowed for comparison to the well described latex of *Hevea* (e.g., dry mass) or promised to reveal key characteristics of the largely unstudied *Campanula* latex. The coagulation and drying speed of *Campanula* latex furthermore is interesting in context of their ecology, hypothesizing that water loss is the main concern of *Campanula* species from more arid regions in case of injury. We also checked for a temperature effect on coagulation and drying time in order to gather insight whether the coagulation mechanism is dominated by chemical or physical processes. Using spectrophotometry, we hoped to observe changes over time during coagulation, that would allow conclusions about chemical processes involved. Bauer et al. have shown *Ficus benjamina* latex to change its IR absorption spectrum during coagulation due to newly appearing covalent amid bonds, for example (Bauer et al. 2010). The wettability experiments were conducted in order to gather information about the hydrophobicity or hydrophily of the dried latex surface. With this initial analysis, we aim to identify differences and similarities between the latex of *Campanula* and the ones of *Hevea* and *Ficus* as a first step toward understanding the coagulation mechanism in *Campanula*.

## Materials and methods

### Plant material

Five specimens of each of the following *Campanula* species were ordered from Staudenhof Menton (https://www.menton-stauden.de/): *C. alliariifolia, C. glomerata, C. lactiflora, C. rapunculoides, C. sarmatica*, and *C. trachelium*. All species used in this study are cultivars, germinated in the year of the study and kept either under greenhouse or garden conditions at the Botanic Garden of the University of Freiburg. Experiments took place from early May through November.

### Coagulation temperature dependency

Coagulation time of different *Campanula* species was determined through semi-quantitative measurements of freshly injured plant specimen. Incisions were made perpendicular to the shoot axis using a razor blade and were deep enough for latex to be discharged. Flowering shoots were preferred for the experiments due to their ample latex discharge, but vegetative shoots or, least favorable, petioles were used in case of shortage. With the flowering and vegetative shoots, incisions up to halfway through the stem were sufficient, but the petioles had to be severed in order to get a sufficient amount of latex. The discharged latex was observed carefully and manipulated in order to identify the change in mechanical behavior, that is, a switch from liquid-like to gel-like behavior. The observation was continued until the latex dried completely in order to compare both coagulation and drying times. For the purpose of validating the coagulation and drying time the experiments were recorded using a CCD camera (digital imaging camera, Panasonic Lumix DMC-FZ1000, Panasonic, Kadoma, Japan). The latex was considered coagulated when the mentioned change in mechanical behavior was observed. Gel-like behavior was characterized by either the latex pulling into strings when manipulated or moving as a bulk of material rather than droplets. It was considered fully dry when its color stabilized (fully transparent), indicating that the sealing process was complete.

The above-described experiments for semi-quantitatively measuring coagulation time were conducted under 3 different temperature set-ups: at room temperature (control, 23.9–25.6°C), on ice (1–2°C), and heated (42–46°C). In the room temperature treatment, latex was observed directly at the incision. That was not possible for the ice and heat treatments since the latex had to be transferred to a chilled or heated object slide, respectively. The object slide for the ice treatment was placed on an aluminum block resting in crushed ice. The object slide for the heat treatment was heated on a heating plate (Medax SP12, Nagel KG, Kiel, Germany) adjusted to 44°C. The following species were analyzed for their latex’ coagulation time under the described temperature treatments: *C. alliariifolia, C. glomerata, C. lactiflora, C. rapunculoides, C. sarmatica*, and *C. trachelium*. The volumes of latex obtained differed per plant species. Coagulation and drying times were determined from the recorded videos by identifying the moment of incision as well as the first proof of coagulation and drying, respectively, as described above. Room temperature and humidity was recorded for each coagulation and drying time experiment individually using a handheld data logger (testo 175-H2, Testo Inc., West Chester, PA, USA).

### Coagulation pressure dependency

In order to test for a pressure dependent coagulation mechanism, as found in *H. brasiliensis* and *F. benjamina*, we tested the *Campanula* species in a custom-built pressure chamber designed by Bauer et al. (2010) for their coagulation time under different pressure regimes. The chamber was equipped with a rotatable blade and a transparent lid. Using 3D printed magnetic sample holders, petioles of *C. alliariifolia* and *C. sarmatica* were positioned inside the chamber in a way that allowed for cutting with the rotatable blade while filming the process from above through the transparent lid. After cutting a single petiole, an acrylic glass shield (attached to the rotatable blade) was used to manipulate the extruding latex in order to determine the moment of coagulation. Measurements were conducted at 8 and 10 bar with both *Campanula* species, 10 repetitions each. The video recordings were used to determine the time of incision and moment of coagulation.

### Spectrophotometry

In preparation for subsequent analytical methods, spectrophotometry was used to verify if diluting *Campanula* latex in distilled water would influence coagulation time. Different volumes of distilled water were used on the latex of the following species: *C. alliariifolia, C. glomerata, C. lactiflora, C. rapunculoides*, and *C. trachelium*. One plant from each species was selected and from each, 3 leaves were severed to harvest latex. Latex from each severed leaf was collected in Eppendorf reaction vials filled with 0.5, 1.0, and 1.5 mL distilled water, respectively. One dilution was selected for each species and was based on prior trials in which we determined latex concentrations that resulted in a measured OD between 0.5 and 1.5 for wavelengths >300 nm. This was done by transferring the loose end of the severed leaf into the reaction vial, wound facing down, and stirring the latex into the distilled water. Since the latex quickly coagulated, its volume was not able to be determined before diluting in distilled water. As a consequence, the final concentration could only be estimated. For the subsequent spectrophotometric analysis, 200 μL were transferred from the upper section of each reaction vial, avoiding latex flocs, onto a 96 well plate (Thermo Scientific™ MaxiSorp coating, Thermo Fisher Scientific Inc., Waltham, MA, USA). In addition, one well on each plate was filled only with distilled water to serve as a control for correcting the raw data. Spectrophotometry was performed using a POLARstar Omega photometer (BMG Labtech, Ortenberg, Germany), with protocol software version 5.10 R2 and firmware version 1.32. After acquiring the data, the spectra were first screened using Mars Data Analysis Software, version 3.10 R6, then the raw data was exported as a spreadsheet in CSV format.

In an initial attempt, the spectrum was recorded once immediately at the start, and then at 30-min intervals for the next 3 h, over a range from 220 to 850 nm with intervals of 2 nm. This measurement protocol was used for samples prepared following the ice, control and heat treatment used in the coagulation and drying time experiments (see coagulation section above). The ice treatment reaction vials were kept on ice during latex dilution and transferring steps. Because the spectrophotometer does not offer any cooling option, the well plates were put on ice between measurements, with a paper napkin underneath to prevent water from fogging the bottom surface. For the heat treatment, the spectrophotometer was set to 45°C during the trial and the well plate was left inside during the measuring period. The control plate was left inside the spectrophotometer during the measurements as well but without a set temperature.

After measurements in 4 species, it was noted that the well plates used for these samples were inaccurate below wavelengths of 280 nm. In a subsequent set of measurements, UV-transparent well plates (Thermo Scientific™Nunc™ line) were used to record spectra of *C. alliariifolia* and *C. sarmatica* using the room temperature treatment set-up. These spectra were recorded in 1-min intervals for 14 min. The change in measurement protocol based on the experiences made with the initially measured 4 species.

### Dry mass

The fresh and dry mass of latex from *C. alliariifolia, C. glomerata, C. lactiflora, C. rapunculoides*, and *C. trachelium* was measured using a fine scale (KERN ABT 220–5DM, KERN & SOHN GmbH, Balingen, Germany). Fresh latex mass was calculated using the equation


(1) \begin{equation*}
{M_{{fresh}}} = {M_{fs}} - {M_s},
\end{equation*}

where ${M_{fs}}$ is the mass of the object slide carrying the fresh latex sample immediately after harvest and ${M_s}$ the mass of the respective pre-weighed object slide. The object slide with the latex sample was dried overnight in an oven at 60°C. Subsequently, the dry mass ${M_{dry}}$ was calculated using the equation


(2) \begin{equation*}
{M_{dry}} = {M_{ds}} - {M_s},
\end{equation*}

where ${M_{ds}}$ is the mass of the object slide carrying the dried latex sample and ${M_s}$ the mass of the object slide. Finally, the dry mass percentage of the fresh latex %*_dry_* was determined using the equation


(3) \begin{equation*}
{{\mathrm{\% }}_{dry}} = \frac{{{M_{dry}}}}{{{M_{{fresh}}}}} \times 100.
\end{equation*}

For all species, we conducted 5 dry mass measurements and calculated the mean dry mass. Due to very low latex harvest, this experiment was altered for the species *C. sarmatica* using pieces of aluminum foil instead of object slides, and a more sensitive fine scale (UMT 2, Mettler Toledo, Columbus, OH, USA).

### Contact angles

Contact angles of 2.5 µL water droplets on coagulated latex were recorded as a measure of wettability. This was done using a custom built setup that allowed for application and lateral video recording of water droplets on a level surface. One side of the setup was a 3D printed platform that could be adjusted along 3 axes to level the sample. This platform was mounted on a micromanipulator. The other side of the setup was a stereo-microscope (Wild Heerbrugg AG, Heerbrugg, Swiss) equipped with a camera (Basler acA2040-90uc, Basler AG, Ahrensburg, Germany). Prior to every measurement, the platform was leveled using a bubble level. Images of the droplets were recorded using “Pylon Camera” Software (Version 7.3.0.27189, Basler AG, Ahrensburg, Germany) and the contact angles of said droplets were subsequently analyzed using the “angle tool” of the software ImageJ (Version 1.54f, Wayne Rasband, NIH, MD, USA). The angles on the right and left side in the picture were measured using this method and the mean was used for further analysis.

The latex films for these measurements were created for each *Campanula* species using the following methods:

(1) The freshly applied latex was spread on an object slide using a razor blade.
(2) The freshly applied latex was spread on an object slide using a razor blade in multiple repetitions.
(3) The fresh latex was applied with the severed plant stem and spread on the object slide in a smooth movement.
(4) The fresh latex was dissolved in 100 µL distilled water before application on the object slide via a pipette.

For all the different applications the latex dried completely before measuring and a minimum of 8 repetitions were conducted per application method.

### Cryo-SEM

Cryo-SEM was used to validate lutoid presence or absence in laticifers of *C. alliariifolia, C. sarmatica, C. glomerata*, and *C. rapunculoides*. Samples were cut from fresh leaf-stems, at least 2 cm from the leaf and approximately 5 mm in length. The samples were attached facing upward to the sample holder using Tissue-Tek O.C.T. Compound, and Aquadag colloidal graphite dissolved in water (both from Plano GmbH, Wetzlar, Germany). Immediately after attachment, the samples were flash-frozen in liquid nitrogen and subsequently transferred into the cryo-chamber (PP310, Quorum Technologies, Laughton, UK) installed on a Zeiss Sigma VP SEM (Carl Zeiss Microscopy, White Plains, NY 10601, USA). The frozen samples were broken using a rotating knife in the cryo-chamber which was kept at −140°C. The broken samples were then transferred into the SEM chamber for sublimation at −85°C for 10 min, followed by platinum sputtering at 30 mA for 90 s. The prepared tissue samples were searched for laticifers and images were recorded using the SE detector at 10 kV in line-average mode (*N* = 15). Laticifers were identified in the resulting images based on their expected position in the collenchyma tissue and literature images for reference (Bauer et al. 2014b).

### Data processing and analysis

R Studio (Version 2022.12.0 + 353 with “R” Version 4.2.2, by the R Core Team) was used to analyze and plot the data. Analysis of variance (ANOVAs) were conducted to test the coagulation data for significant influence of treatment (ice, control, and heat) as well as for room temperature and humidity on the coagulation and drying time of the different species. One ANOVA was conducted, each for coagulation and drying time, for independent effects of the variables “plant species” and “treatment,” as well as for independent and combined effects of the variables “room temperature” and “room humidity.” Furthermore, ANOVA's were used to identify significant differences between the contact angles for different surface application methods of the latex. Bonferroni-corrected pairwise-tests were used to identify significant differences between the individual treatments if the ANOVA revealed any. The coagulation time under different pressures was tested for significant differences using Kruskal–Wallis tests in combination with continuity corrected pairwise Wilcoxon tests.

## Results

### Coagulation temperature dependency

**Fig. 1.**
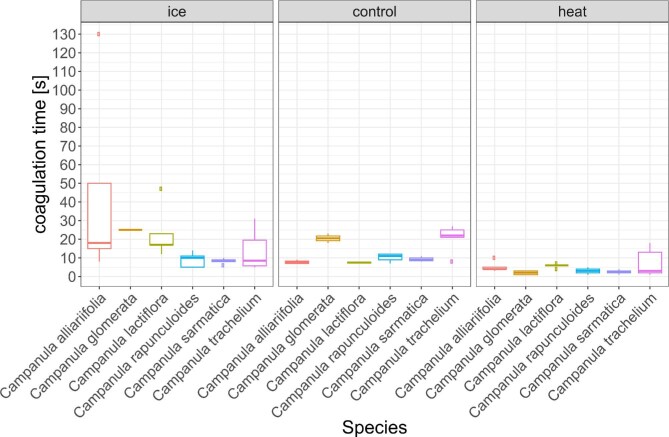
Latex coagulation time in in seconds for *C. alliariifolia, C. glomerata, C. lactiflora, C. rapunculoides, C. sarmatica*, and *C. trachelium* for 3 different temperatures treatments (“ice” = 1–2°C; “control” = 23.9–25.6°C; “heat” = 42–46°C).

We observed latex coagulation times of about 13 s on average across species at room temperature and about 19 s on average across species on ice (mean coagulation time ice: 19.2 s, SD = 24.6, control: 12.8 s, SD = 6.7). As a reference, *F. benjamina* latex has been reported to take 20 min to coagulate (Bauer et al. 2010), *H. brasiliensis* latex 30 min, and more (Gidrol et al. 1994). The coagulation times in the heat treatment were shorter in comparison to the control treatment (mean coagulation time heat: 4.3 s, SD = 3.7, Fig. 1 and S-Table 1). A Shapiro-test confirmed normal distribution and an ANOVA revealed the influence of treatment as significant (*df* = 2, *F* = 7.602, *P* < 0.005). However, only the heat treatment showed significant differences compared to the other treatments, and not in every species (Tukey's test): *C. glomerata* and *C. sarmatica* show significant differences between heat and ice, and heat and control treatments (*P* < 0.001), *C. lactiflora* and *C. rapunculoides* both show a significant difference between heat and ice (*P* < 0.05), and *C. rapunculoides* also shows a significant difference between heat and control (*P* < 0.005). *Campanula alliariifolia* and *C. trachelium* showed no significant differences between any of the treatments. The variation in room temperature did not affect coagulation time, while the ANOVA revealed humidity to have an effect on coagulation time. Datapoints recorded under high humidity of above 70% displayed above average coagulation times.

**Fig. 2.**
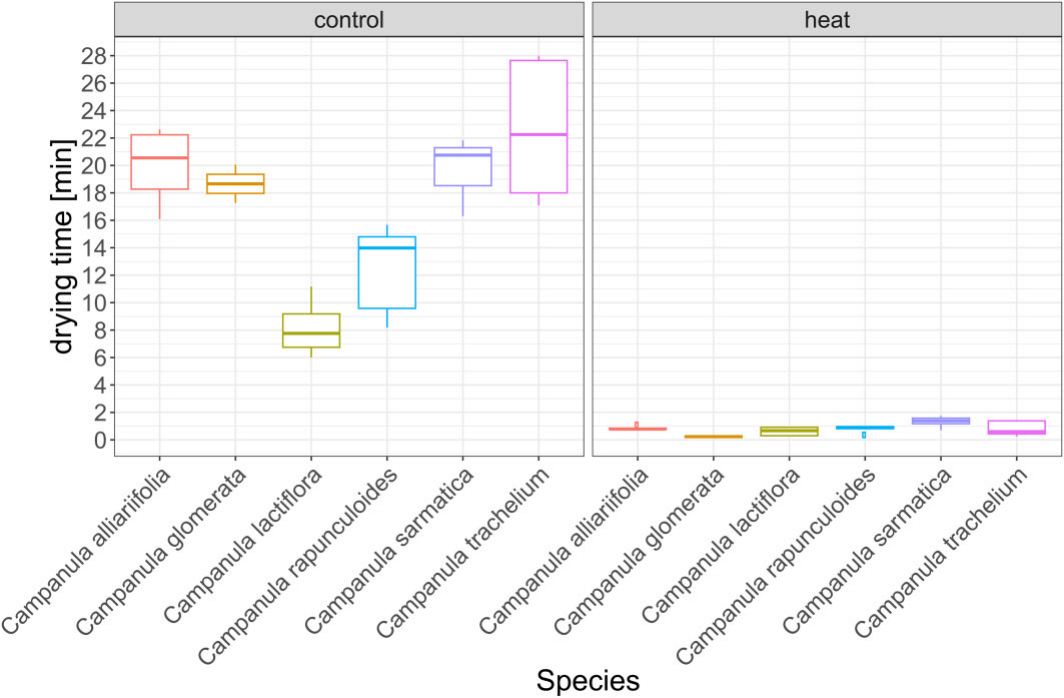
Latex drying time in minutes for *C. alliariifolia, C. glomerata, C. lactiflora, C. rapunculoides, C. sarmatica*, and *C. trachelium* for different temperatures treatments (“control” = 23.9–25.6°C; “heat” = 42–46°C). Condensation prevented drying in the “ice” treatment.

Latex drying time in the heat treatment was significantly lower compared to the control treatment in all species (mean drying time control: 12.81 min, SD = 6.75, heat: 4.26 min, SD = 3.68, Fig. 2 and S-Table 2). An ANOVA confirmed significant differences between treatments (*df* = 1, *F* = 268.997, *P* < 0.001) as well as between the species (*df* = 5, *F* = 5.805, *P* < 0.001). The ice treatment was excluded from the analysis, because condensation on the object slide prevented the latex from drying. Neither ambient temperature nor humidity proved to influence the drying time of the latex.

### Coagulation pressure dependency

**Fig. 3.**
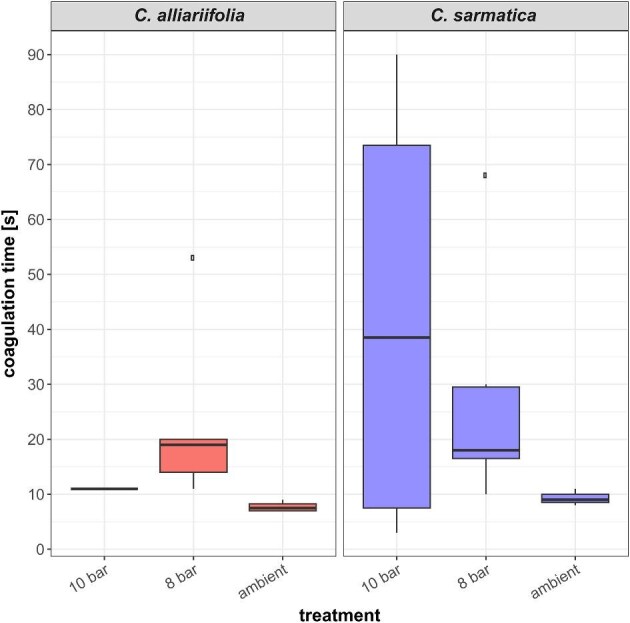
Coagulation time in seconds for *C. alliariifolia* and *C. sarmatica* measured at room temperature in a pressure chamber under 3 different pressures: atmospheric, 8 bar, and 10 bar.

The coagulation time measured at ambient pressure for the 2 species *C. alliariifolia* and *C. sarmatica* were 7.5 s median and 9 s median, respectively. The measured coagulation times at 8 bar were 19 s median for *C. alliariifolia* and 18 s median for *C. sarmatica.* At 10 bar, the measured coagulation times were 11 s for *C. alliariifolia* and 38.5 s for *C. sarmatica* (Fig. 3). Bauer et al. reported a noticeable change in latex coagulation time for *F. benjamina* when injured in pressure regimes of 2 bar and above. Under high pressure instead of 20 min, several hours were necessary for the latex to coagulate (Gidrol et al. 1994). Kruskal–Wallis tests and continuity corrected pairwise Wilcoxon were conducted to identify significant differences between the treatments. The Kruskal–Wallis test for *C. alliariifolia* indicated significant differences between the treatments (Kruskal–Wallis test *C. alliariifolia*: *df* = 2, chi-squared = 7.1558, *P* = 0.02793, Kruskal–Wallis test *C. sarmatica*: *df* = 2, chi-squared = 3.1492, *P* = 0.2071) but the subsequent post-hoc analysis did not confirm any significant difference (pairwise Wilcoxon test ambient vs. 8 bar: *P* = 0.058).

### Spectrophotometry

**Fig. 4.**
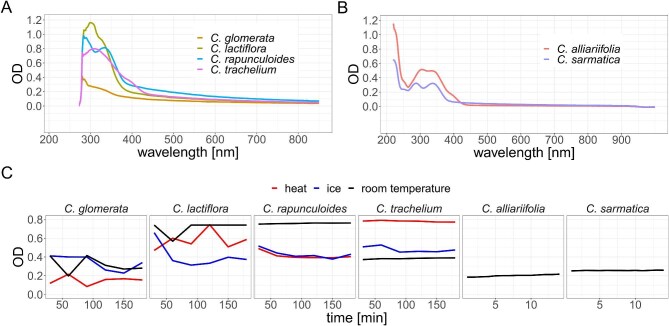
Spectrophotometric analysis of different *Campanula* species’ latex coagulation, plotted as optic density (OD) as a function of wavelength in nm (**A, B**) or time in seconds (**C**). (**A**) Photometric spectra of latex diluted in different volumes of distilled water at room temperature, wavelength 270–850 nm: *C. glomerata* in 0.5 mL, *C. lactiflora* in 1.5 mL, *C. rapunculoides* in 0.5 mL, and *C. trachelium* in 1.0 mL. (**B**) Photometric spectra of latex diluted in different volumes of Aqua dist. at room temperature, wavelength 220–1000 nm using a UV transparent well plate: *C. alliariifolia* in 3.0 mL, *C. sarmatica* in 1.5 mL. (**C**) OD of different *Campanula* species’ latex solutions in distilled water over time at specific wavelengths. Volumes of distilled water as well as observed wavelengths were optimized for every species individually: for the used volumes of distilled water see (**A**) and (**B**), the species-specific wavelengths were chosen where spectra expressed changes over time: *C. glomerata*: 280 nm, *C. lactiflora*: 280 nm, *C. rapunculoides*: 312 nm, *C. trachelium*: 388 nm, *C. alliariifolia*: 388 nm, and *C. sarmatica*: 312 nm. *Campanula alliariifolia* and *C. sarmatica* were analyzed using a different protocol including UV transparent well plates, a shorter observation period, higher measurement frequency and was conducted only at room temperature.

The recorded photometric spectra show a number of similarities throughout all tested species. In *C. sarmatica, C. lactiflora, C. rapunculoides*, and *C. trachelium*, we observed absorption between wavelength ∼280 and ∼440 nm (Fig. 4A). Both *C. glomerata* and *C. rapunculoides* show a maximum optical density at approximately 280 nm. However, while *C. glomerata*’s optical density drops quickly from 0.4 to 0.3 OD at ca. 295 nm and then declines slowly afterward, *C. rapunculoides* shows a second local maximum at 340 nm. This second local maximum is lower (∼0.8 OD) than the first one (∼1 OD) and is separated from it by a local minimum (∼0.75 OD) at ca. 315 nm. After this second maximum, the optical density follows a steep drop to ∼0.3 OD at ca. 380 nm and declines steadily from there with increasing wavelength. *Campanula lactiflora* and *C. trachelium* show one local maximum each that are at longer wavelengths than in *C. glomerata* and *C. rapunculoides*. The local maximum in the spectrum of *C. lactiflora* is found at 300 nm, that of *C. trachelium* at ∼320 nm. The optical density drops to lower values after the respective local maxima following different patterns in *C. lactiflora* and *C. trachelium*. In *C. lactiflora*, it follows a steep drop with a minor shoulder at ∼325 nm (∼0.95 OD), while in *C. trachelium*, the optical density drops more steadily with increasing wavelength, showing a broader shoulder at ca 390 nm (∼0.38 OD). The spectra of the observed species revealed values tending toward zero from wavelength ∼370 nm and up, with the exception of *C. trachelium* (∼460 nm). The 2 species *C. alliariifolia* and *C. sarmatica* were measured in well plates suited for measurements in the UV spectrum (Fig. 4B). The high values and subsequent steep decline at wavelength ∼230 nm to ∼260 nm are caused by the well plates, as documented by the manufacturer. The spectra of *C. alliariifolia* and *C. sarmatica* appear very similar as they both show 2 local maxima followed by a steep drop in optical density. While the first local maximum is at ca. 280 nm in *C. sarmatica*, it is at a higher wavelength in *C. alliariifolia* (300 nm). A second apparent difference is that the local minimum in between the 2 local maxima shows a higher difference in value to the maxima in *C. sarmatica* (∼0.06 ΔOD) than in *C. alliariifolia* (∼0.01 ΔOD). Both species’ spectra display a second local maximum at ∼380 nm. The spectrum of *C. alliariifolia* shows a shoulder at ∼380 nm, the one of *C. sarmatica* does not. Instead, the spectrum of *C. sarmatica* shows a tendency toward zero from ∼380 nm toward longer wavelengths, *C. alliariifolia* shows this tendency starting at ∼425 nm. The high OD in all our *Campanula* latex samples at around 280 nm wavelength seems noteworthy as Archer reported an absorption maximum for Hevein at 280 nm wavelength (Archer 1960).

All observed species revealed time dependent differences in their respective spectra. For each species, the wavelength showing the biggest changes over time was determined and for these wavelengths, the OD was plotted over time (Fig. 4C). The respective wavelength represented different spectrum characteristics for the different species: local maximum (*C. glomerata*: 280 nm, *C. lactiflora*: 280 nm), local minimum (*C. rapunculoides*: 312 nm, *C. sarmatica*: 312 nm), and shoulder (*C. trachelium*: 388 nm, *C. alliariifolia*: 388 nm). For the species *C. glomerata, C. lactiflora, C. rapunculoides*, and *C. trachelium*, spectra were recorded over time at different temperatures according to the protocols for coagulation- and drying-time. *Campanula alliariifolia* and *C. sarmatica* spectra were recorded only at room temperature over time. In *C. glomerata* and *C. lactiflora*, a decline of OD was observed in the ice treatment whereas the room temperature and heat treatment did not exhibit an upward or downward trend but showed high variation. *Campanula rapunculoides* and *C. trachelium* showed stable OD in the room temperature treatment and a small decline of OD in both the heat and ice treatment. *Campanula alliariifolia* showed a small increase of OD at room temperature, while in *C. sarmatica*, the OD remained stable.

### Dry mass

The dry mass percentage varied between the species, as can be seen in Table 1. The lowest dry mass was observed in *C. rapunculoides* (12.2%). The other species showed higher latex dry mass as follows, in order of increasing dry mass: *C. trachelium* (16.9%), *C. sarmatica* (20.3%), *C. glomerata* (25.7%), *C. alliariifolia* (27.6%), and *C. lactiflora* (39.7%).

**Table 1.** Calculated latex dry mass percentage of *C. alliariifolia, C. glomerata, C. lactiflora, C. rapunculoides, C. sarmatica*, and *C. trachelium*, as well as literature value for *H. brasiliensis* (d'Auzac and Jacob 1989)

**Species**	**Latex dry mass [%]**	**SD**	***n***
*C. alliariifolia*	27.6	6.3	5
*C. glomerata*	25.7	23.1	5
*C. lactiflora*	39.7	7.8	5
*C. rapunculoides*	12.2	6.4	5
*C. trachelium*	16.9	8.4	5
*C. sarmatica*	20.3	3.5	5
*H. brasiliensis*	40		

### Contact angles

**Fig. 5.**
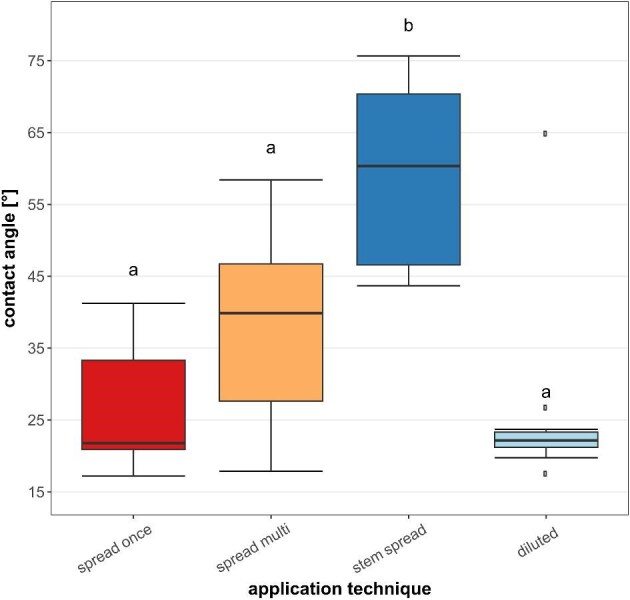
Contact angles of 2.5 µL water droplets on *C. alliariifolia* latex films, produced with different techniques.

The measured contact angles of water drops on latex film showed high deviation for all tested application techniques (spread once: mean = 26.97°, SD = 9.00; spread multi: mean = 37.88°, SD = 12.00; stem spread: mean = 59.17°, SD = 13.15; diluted: mean = 25.89°, SD = 13.13, Fig. 5). ANOVA testing with subsequent post-hoc test showed the differences between “stem spread” and all other used techniques to be significant (ANOVA, *df* = 3, *F* = 14.75, *P* < 0.005; Tukey's-test)

### Cryo-SEM

**Fig. 6.**
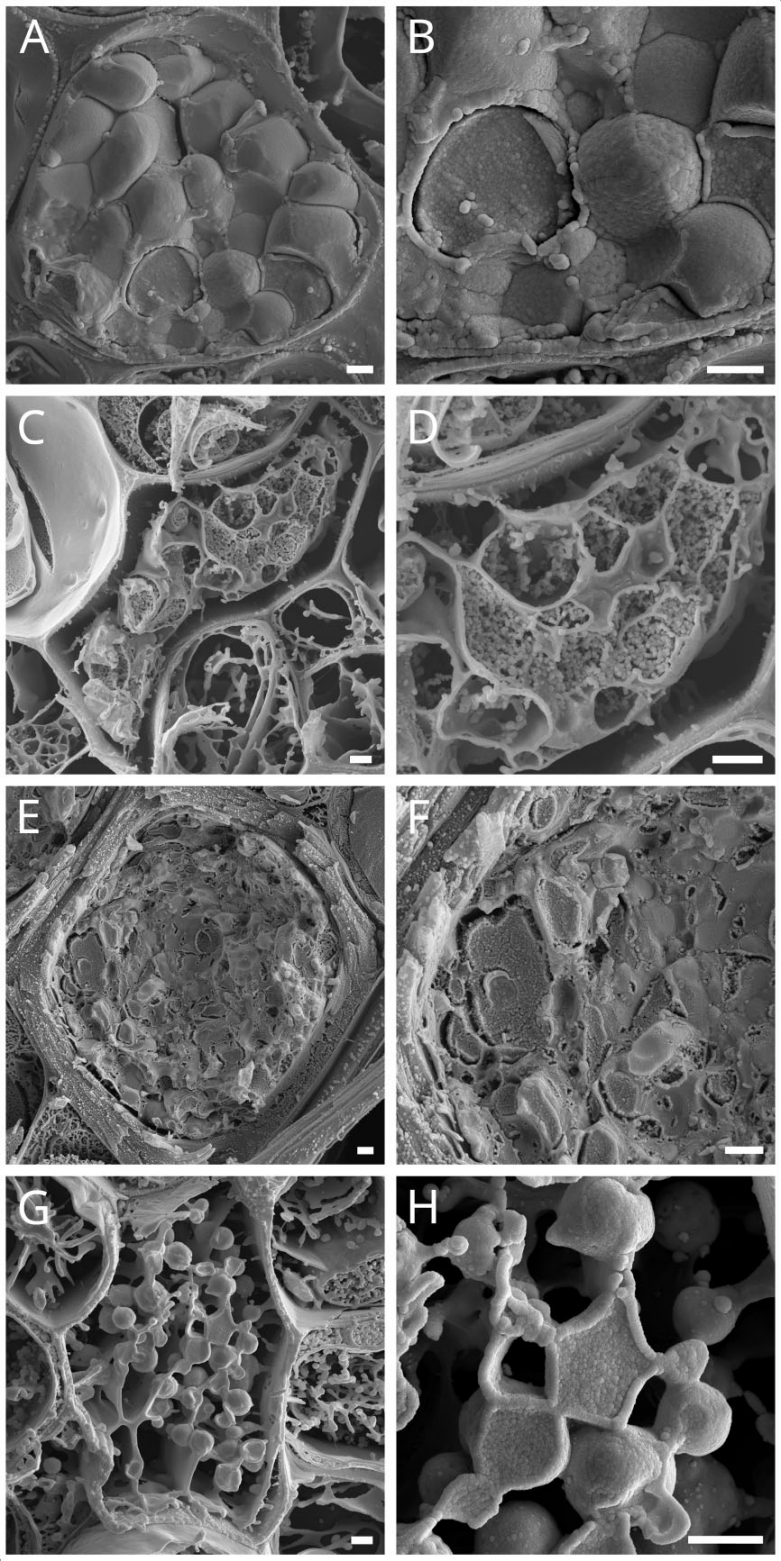
Cryo-SEM images of *C. alliariifolia* (**A, B**), *C. sarmatica* (**C, D**), *C. glomerata* (**E, F**), and *C. trachelium* (**G, H**). For each species, an overview of a transection cut through a laticifer is shown (**A, C, E, G**) as well as a close-up depicting the internal structures (**B, D, F, H**). Scale bars = 1 µm.

**Fig. 7.**
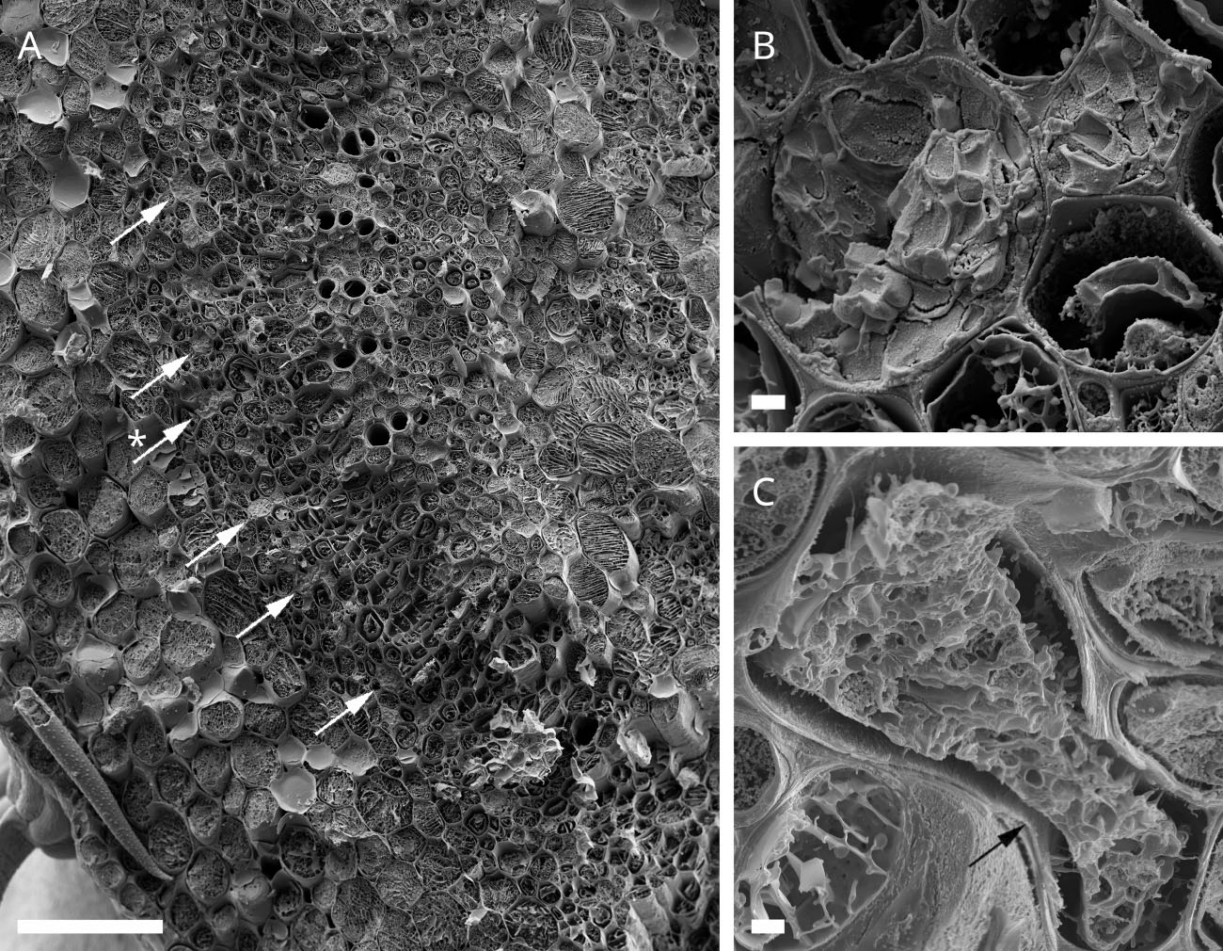
Cryo-SEM images of laticifers in *Campanula.* (**A**) Cross section of *C. alliariifolia* petiole. White arrows indicate position of laticifers, indicating their distribution in the collenchyma tissue. Scale bar = 10 µm. (**B**) Magnification of 2 laticifers labeled with asterisk over a white arrow in (**A**). Scale bar = 2 µm. (**C**) Bifurcating laticifer of *C. sarmatica*, the black arrow indicates bifurcation area. Scale bar = 2 µm.

Using Cryo-scanning-electron-microscopy, we recorded images of laticifers of *C. alliariifolia* (Fig. 6A and B), *C. sarmatica* (Fig. 6C and D), *C. glomerata* (Fig. 6E and F), and *C. trachelium* (Fig. 6G and H). Figure 6 displays cross section overviews of the laticifer in the respective species (A, C, E, and F) as well as a higher magnification of the latex structures (B, D, F, and H). In all species, the laticifers were found in the collenchyma, evenly scattered between vascular bundles and parenchymatic cells of the cortex (Fig. 7A and B). In some of the samples, we were able to observe bifurcated laticifers (Fig. 7C). All species showed a similar structure in their latex, while the density in the laticifers, that is, the amount of latex found in the respective cells, differed between samples and species. The latex appeared to consist of small round units of about 0.1 µm that conglomerate to bigger structures of oval or round shapes with diameters of 0.5–1.5 µm. These bigger structures appear to interconnect via bridges made of the round units in a web-like manner. We did not identify any other cell organelles in the laticifers in our sample.

## Discussion

Our coagulation and drying time data confirm that *Campanula* coagulates quickly, as noted in an earlier publication (Bauer et al. 2014b). We found all studied species to have coagulation times in less than a minute; the majority of species displayed latex coagulation times of less than 10 s on average. We found this coagulation time to be temperature dependent, as our heat treatment (42–46°C) sped up the process significantly for half of the species (*C. glomerata, C. rapunculoides*, and *C. sarmatica*) when compared to the control treatment. Interestingly, none of the species showed a significantly reduced coagulation time in the ice treatment (1–2°C) compared to the room temperature treatment. In comparison to the heat treatment, the ice treatment had a significantly prolonged coagulation time in 4 species (*C. glomerata, C. lactiflora, C. rapunculoides*, and *C. sarmatica*). While it is not surprising that temperature plays a role in the coagulation process, it is noteworthy that some species’ latex (e.g., *C. glomerata*) appears to be more influenced by it in terms of coagulation speed and in *C. alliariifolia* the variation was very different between the temperature treatments. In the ice treatment, *C. alliariifolia* showed coagulation times ranging from 10 s to 2 min. A recent study by Langhoff et al. (2025), in fact, provides evidence that coagulation in *Campanula* latex happens as a chemical reaction in the bulk material. These new findings offer some explanation for why coagulation is so fast in *Campanula* as film formation, evaporation, or surface related reactions like e.g., oxidation would be much slower than the observed coagulation times. A reaction in the bulk material, however, still would be influenced by temperature, like our experiments show.

The drying time data provide a clearer picture of the influence of temperature. In all species, the heat treatment significantly reduced the latex drying time to about 1 min and sometimes below. The very short drying time and low variation between and within the species is most likely due to the increased evaporation in the heat treatment. The latices’ fast loss of water through evaporation in the heat treatment is likely to dominate any other temperature driven effect in the drying process. The latex drying time at room temperatures shows quite high variation within, as well as in between species. This variation is a consequence of the amount of latex measured in each experiment. The species we observed to be richest in latex, and therefore with the largest amount per measurement, clearly show the longest drying time (*C. alliariifolia, C. glomerata*, and *C. sarmatica*), while the one producing the smallest amount of latex (*C. lactiflora*) shows the shortest drying times. Due to the constant condensation on the object slide surface and the respective latex sample during the ice treatment, we were not able to determine drying time for low temperatures on ice.

In contrast to the coagulation of *Ficus* latex, *Campanula* latex does not slow down its coagulation significantly under increased pressure (Bauer et al. 2010). The high deviation in our data is caused by handling challenges and rather reflect a minimum handling time before recording the data rather than an actual coagulation time measurement. Even though our set-up did not allow for a consistently fast measurement, the data clearly show coagulation times well under a minute in all tested pressure regimes and therefore strongly supports a different coagulation mechanism in *Campanula* than in *Hevea* and *Ficus*.

The majority of the absorbance (or scattering) for all analyzed species was found at wavelengths between 260 and 420 nm. Interestingly, we observed changes over time that cannot be explained by evaporation, since these changes are restricted to specific wavelengths within the range where we observed absorbance. Furthermore, these changes in OD over time were observed in rather narrow wavelength ranges, while the remaining spectrum displayed stable OD values over time. We interpret these changes as coagulation-related processes. One of the wavelength ranges that regularly displayed OD changes over time in the analyzed species was around 280 nm, a wavelength that is commonly used to determine protein content of liquid samples, indeed Archer also reported an absorption maximum for Hevein at 280 nm (Bauer et al. 2010). It is known that latices contain high numbers and amounts of proteins (Konno 2011) and it is not unlikely that proteins involved in coagulation, for example, connecting latex particles, change their conformation during coagulation, resulting in different absorbance properties. Bauer et al. (2010), for example, were able to link changes in IR absorption over time observed in coagulating latex of *Ficus benjamina* to the formation of new covalent amid bonds. Furthermore, the mere conglomeration of particles, that is, coagulation, causes light scattering and subsequently changes absorbance of the sample. In *Hevea*, for example, Hevein has shown to change absorption of latex dilutions at 600 nm wavelengths; as coagulation commences the absorption values decrease as the latex particles conglomerate (Gidrol et al. 1994). In sum, our spectrophotometry data hint toward a chemical reaction during latex coagulation in *Campanula* that includes protein binding or restructuring as absorption at UV wavelengths that are suitable for measuring protein content change over time in our samples. The fact that Hevein absorbs at 280 nm, where we find high optical density in our samples, has to interpreted conservatively. It appears unlikely that *Campanula* latex includes Hevein, as this protein has been only reported for *Hevea* and many proteins absorb in this wavelength spectrum. Nonetheless, it is possible that *Campanula* latex includes proteins that are similar to Hevein and thus show similar UV absorbance behavior. This is, however, to be clarified in subsequent studies.

Even though our experiences gathered during the spectrophotometric analysis taught us that the latex poorly disperses in water, it still shows hydrophilic characteristics when dried. Our contact angle measurements showed high deviation within the different surface application methods of the latex as well as between them, but all measured angles were below 90° and therefore in the hydrophilic spectrum. The average contact angles differed remarkably between the application techniques, which might be due to mechanical destruction of particles on the surface, revealing their inner hydrophobic core. With increasing mechanical stress caused by the application method, the likelihood increases to brake open the particles and exposing the long-chained, hydrophobic rubber polymer the particles consist of. Our observation might also be a combination of this discussed chemical and physical surface effects. Spreading the fresh latex on an object slide via a razor edge or the cutting edge of the stem it originates from, certainly makes a difference in how smooth the surface one creates will be. The high contact angles measured on the latter surface thus are probably due to grooves left behind from the uneven stem surface. In fact, this hypothesis is supported by the application method “diluted,” where latex was first diluted and then applied as a droplet and subsequently dried, undoubtedly resulting in the smoothest surface of all tested techniques. With the exception of one outlier, the data for this application technique in comparison to the other ones is remarkably narrow and ranges among the lowest contact angles measured (see Fig. 5).

Our observed range of dry mass (12–40%) in the analyzed *Campanula* species include dry mass values that are comparable to fresh *Hevea* latex (∼40%) (d'Auzac and Jacob 1989). A correlation between latex dry mass and coagulation- or drying time of the respective species was not observable. While *C. lactiflora* latex, dry mass of 39.7%, did dry fastest, its coagulation time was among the slower species. *Campanula rapunculoides* latex, dry mass of 12.2%, coagulated rather fast but its drying time was second fastest. We therefore cannot confirm an effect of latex dry mass on neither coagulation nor drying time. The dry mass variation within and between *Campanula* species can be due to a number of reasons. Foremost, the amount of latex harvested and measured was in general pretty low and thus the precision of the used fine scale added to the variation observed. Furthermore, water potential in the plant may have influenced water content of latex and therefore the dry mass measurements. Since we observed not only different dry masses but also different amounts of latex in the different species, it is also safe to assume that some of this variation is due to their respective ecology. Differences in the respective ecology might include predator/parasite pressure, natural habitat's climate or water accessibility. *Campanula lactiflora*, for example, with the smallest amounts of latex produced and simultaneously the highest latex dry mass, is native to Turkey and the Caucasus where dry to arid climates dominate.

Our Cryo-SEM analysis furthermore adds to the overall picture that latex coagulation in *Campanula* is different to that observed in *Hevea* and *Ficus.* Prior studies have shown both solid (latex) particles and membranous lutoids, that contain the Hevein/Hevein-like proteins in the latices of *Ficus* and *Hevea* (Bauer et al. 2014b; Ng et al. 2022). We did not find any latex particles of comparable size to *Ficus* or *Hevea* in *Campanula*, nor did we find any structure resembling the described membranous lutoids. In fact, in *Campanula* laticifers, we found densely packed round structures of about 0.1 µm diameter, conglomerating to bigger (∼0.5–1 µm average diameter), interconnected structures. Given the smaller latex particles, not accompanied by lutoids, the *Campanula* latex somewhat resembles the one described in *Euphorbia* species (Bauer et al. 2014b). The particles in *Campanula* are even smaller though than the ones in *Euphorbia* (0.1 and ∼0.2 µm, respectively) and the coagulation times in *Campanula* are considerably faster than the ones in *Euphorbia*. The extremely short time needed for coagulation in *Campanula* spp. excludes a pure evaporation effect as in *Euphorbia* spp. as (main) driving force for the coagulation process. *Campanula*’s latex organization gives the impression of somehow phase-separated highly viscous material in the cell plasma. As there is no visible compartmentalization in the laticifers, it furthermore leaves the question of how the latex is prevented from coagulating within the laticifers and what the mechanism and trigger of coagulation after injury might be.

## Conclusion

Our findings demonstrate that the latex found in *Campanula* behaves considerably different from well-studied systems like *Hevea, Ficus*, and *Euphorbia.* Not only is the coagulation of *Campanula* latex remarkably faster, but it also does not slow down under pressures of 8 bar and above. Our Cryo-SEM images furthermore reveal noteworthy differences to latices of *Hevea* and *Ficus.* In contrast to those well-studied systems, the *Campanula* latex seems not to consist of colloid latex particles (like *Hevea, Ficus*, and *Euphorbia*) forming an emulsion with membranous lutoid particles in the laticifer cytoplasm, like it is found in *Hevea* and *Ficus*, but rather be an emulsion of highly viscous latex droplets within the laticifer cytoplasm. Despite the similarities found, that is, dry mass of some *Campanula* species comparable to the one reported for *Hevea* and high absorbance in *Campanula* latex at wavelengths reported for Hevein, our data clearly indicates a different coagulation mechanism in *Campanula* than described for *Hevea*. This different coagulation mechanism and the different latex characteristics may have evolved from different ecological constraints, as both *Ficus* and *Hevea* are found in tropical habitats whereas *Campanula* is prevalent in temperate and arid climates of Eurasia. The distribution in arid climate as well as the latex particle size are traits that *Campanula* shares with the *Euphorbia* species originally studied by Bauer et al. (2014b). However, despite the similarities to *Euphorbia, Campanula* coagulates remarkably faster than *Euphorbia*, which therefore likely follows a different strategy for preventing water loss. *Campanula*’s mechanism of latex coagulation and how the plant prevents its onset until injury, yet remain to be discovered.

## Supplementary Material

obaf020_Supplemental_File The supplementary data contains three tables and one figure. S-Table 1 and S-Table 2 list data plotted in Fig. 1 and Fig. 2, while S-Table 3 contains details of all conducted ANOVAs. S-Figure 1 gives example frames from our semi-quantitative coagulation experiments that represent different states of *Campanula* coagulation, that is, “liquid” vs. “coagulated” vs. “dried.”

## Data Availability

The data underlying this article are available in the article and in its online supplementary material.
