# Supplementary material for: Toward understanding the fast latex coagulation in Campanula spp. (Campanulaceae)
Source: Integr Org Biol. 2025 May 14;7(1):obaf020. doi: 10.1093/iob/obaf020 (PMC12120442; doi:10.1093/iob/obaf020)
Supplement: obaf020_Supplemental_File — The supplementary data contains three tables and one figure. S-Table 1 and S-Table 2 list data plotted in Fig. 1 and Fig. 2, while S-Table 3 contains details of all conducted ANOVAs. S-Figure 1 gives example frames from our semi-quantitative coagulation experiments that represent different states of Campanula coagulation, that is, “liquid” vs. “coagulated” vs. “dried.” [file obaf020_supplemental_file.docx]

Supplementary Materials for

Towards understanding the fast latex coagulation in *Campanula* spp. (Campanulaceae)

Maartje H.M. Wermelink^1,2^, Merle L. Becker^1^, Rupert Konradi^3,4^, Claude Taranta^5^, Meik Ranft^4^, Simon Nord^5^, Jürgen Rühe^2,3,6^, Thomas Speck^1,2,3^, Sebastian Kruppert^1,2,3,*^

1. Plant Biomechanics Group @ Botanical Garden, University Freiburg, Freiburg im Breisgau, Germany
2. Excellence Cluster *Liv*MatS, University Freiburg, Freiburg im Breisgau, Germany
3. Joint Research Network on Advanced Materials and Systems (JONAS), Freiburg Materials Research Center, University Freiburg, Freiburg im Breisgau, Germany
4. BASF SE, Group Research, Joint Research Network on Advanced Materials and Systems (JONAS), Ludwigshafen, Germany
5. BASF SE, Agricultural Solutions, Limburgerhof, Germany
6. IMTEK, University Freiburg, Freiburg im Breisgau

ORCIDs:
M.H.M Wermelink: 0009-0000-2337-6340
T. Speck: 0000-0002-2245-2636
S. Kruppert: 0000-0001-5932-8455

*Corresponding author: Sebastian Kruppert

Georges-Köhler-Allee 105, 79106 Freiburg im Breisgau,

email: sebastian.kruppert@biologie.uni-freiburg.de

phone: +49 761 203 2781

**The PDF file includes:**

S-Table 1 – S-Table3

S-Figure 1

**S-Table 1:** Mean latex coagulation time in seconds in *C. alliariifolia, C. glomerata, C. lactiflora, C. rapunculoides, C. sarmatica* and *C. trachelium* for three different temperatures.

| **Species** | **Treatment** | **Mean coagulation**  **time [s]** | **SD** | **n** |
| --- | --- | --- | --- | --- |
| *Campanula alliariifolia* | Control | 7.8 | 1.0 | 4 |
|  | Heat | 5.2 | 2.8 | 5 |
|  | Ice | 44.2 | 50.6 | 5 |
| *Campanula glomerata* | Control | 20.5 | 3.5 | 2 |
|  | Heat | 2.0 | 1.0 | 5 |
|  | Ice | 25.0 | *NA* | 5* |
| *Campanula lactiflora* | Control | 7.5 | 0.7 | 4 |
|  | Heat | 5.8 | 1.1 | 5 |
|  | Ice | 23.2 | 13.8 | 6 |
| *Campanula rapunculoides* | Control | 10.2 | 2.2 | 5 |
|  | Heat | 3.0 | 1.6 | 5 |
|  | Ice | 9.0 | 3.9 | 5 |
| *Campanula sarmatica* | Control | 9.3 | 1.5 | 3 |
|  | Heat | 2.5 | 1.0 | 6 |
|  | Ice | 8.3 | 1.4 | 6 |
| *Campanula trachelium* | Control | 20.6 | 7.4 | 5 |
|  | Heat | 7.4 | 7.6 | 5 |
|  | Ice | 13.5 | 10.9 | 6 |
| **All species mean and total n** | Control | 12.8 | 6.7 | 23 |
|  | Heat | 4.3 | 3.7 | 31 |
|  | Ice | 19.2 | 24.6 | 33 |

*Only one sample allowed for a measurement, therefore this value is not representative.

**S-Table 2:** Mean latex drying time in minutes in *C. alliariifolia, C. glomerata, C. lactiflora, C. rapunculoides, C. sarmatica* and *C. trachelium* for three different temperatures.

| **Species** | **Treatment** | **Mean drying**  **time [min]** | **SD** | **n** |
| --- | --- | --- | --- | --- |
| *Campanula alliariifolia* | Control | 19.95 | 3.04 | 4 |
|  | Heat | 0.84 | 0.15 | 5 |
|  | Ice | *NA* | *NA* | 5 |
| *Campanula glomerata* | Control | 18.66 | 1.97 | 2 |
|  | Heat | 0.23 | 0.06 | 5 |
|  | Ice | *NA* | *NA* | 5 |
| *Campanula lactiflora* | Control | 8.17 | 2.25 | 4 |
|  | Heat | 0.62 | 0.32 | 5 |
|  | Ice | *NA* | *NA* | 6 |
| *Campanula rapunculoides* | Control | 12.44 | 3.34 | 5 |
|  | Heat | 0.80 | 0.26 | 5 |
|  | Ice | *NA* | *NA* | 5 |
| *Campanula sarmatica* | Control | 19.63 | 2.93 | 3 |
|  | Heat | 1.33 | 0.38 | 6 |
|  | Ice | *NA* | *NA* | 6 |
| *Campanula trachelium* | Control | 22.60 | 5.16 | 5 |
|  | Heat | 0.82 | 0.55 | 5 |
|  | Ice | *NA* | *NA* | 6 |
| **All species mean and total n** | Control | 12.81 | 6.75 | 23 |
|  | Heat | 4.26 | 3.68 | 31 |
|  | Ice | *NA* | *NA* | 33 |

**S-Table 3:** ANOVA results for latex coagulation time, latex drying time and contact angles of water droplets on latex film.

| **ANOVA** | **Variable** | **Df** | **Sum sq** | **Mean Sq** | **F-value** | **p-value** |
| --- | --- | --- | --- | --- | --- | --- |
| **coagulation time** | Species | 5 | 1805 | 360.9 | 1.682 | 0.15061 |
|  | Treatment | 2 | 3263 | 1631.6 | 7.602 | 0.00104 ** |
|  | Humidity | 1 | 900 | 899.7 | 4.192 | 0.04443 * |
|  | Room Temperature | 1 | 185 | 184.5 | 0.860 | 0.35702 |
|  | Humidity:Room temperature | 1 | 18 | 18.2 | 0.085 | 0.77192 |
|  | Error | 69 | 14809 | 214.6 |  |  |
| **drying time** | Species | 5 | 355 | 71 | 5.805 | <0.0005 *** |
|  | Treatment | 1 | 3288 | 3288 | 268.997 | <0.00001 *** |
|  | Humidity | 1 | 0 | 0 | 0.003 | 0.954 |
|  | Room Temperature | 1 | 6 | 6 | 0.460 | 0.501 |
|  | Humidity:Room temperature | 1 | 4 | 4 | 0.350 | 0.557 |
|  | Error | 44 | 538 | 12 |  |  |
| **contact angle** | film technique | 3 | 6290 | 2096.8 | 14.75 | <0.00001 *** |
|  | Error | 43 | 6113 | 142.2 |  |  |

**
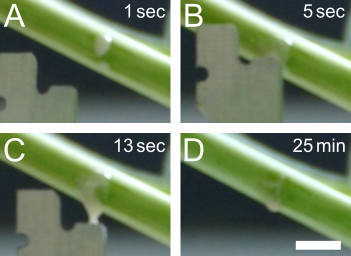
**

**S-Figure 1**: Latex on a fresh incision of a C. sarmatica petiole. **A** Fresh drop of latex discharging one second after incision. **B** Fresh latex (5 seconds after incision) still exhibiting liquid-like behavior, i.e. no stringing or plastic deformation of the droplet. The whole volume of latex is drawn to the blade as effect of adhesion. **C** Coagulated latex exhibiting gel-like behavior (13 seconds after incision). The bulk of the volume stays in place while part of the latex strings due to adhesion to the blade. **D** Dried latex, 25 minutes after incision. Areas where the latex covers the petiole in a thinner layer appear transparent; more voluminous areas are milky yellow. Scale bar ≈ 5 mm.
